# Recombination of the porcine X chromosome: a high density linkage map

**DOI:** 10.1186/s12863-014-0148-x

**Published:** 2014-12-20

**Authors:** Ana I Fernández, María Muñoz, Estefânia Alves, Josep María Folch, Jose Luis Noguera, Miguel Pérez Enciso, Maria del Carmen Rodríguez, Luis Silió

**Affiliations:** Departamento de Mejora Genética Animal, INIA, Ctra. De la Coruña km. 7, Madrid, 28040 Spain; The Roslin Institute and R(D)SVS, University of Edinburgh, Midlothian, EH25 9RG UK; Departament de Ciència Animal i dels Aliments, Facultat de Veterinària, UAB, Bellaterra, 08193 Spain; Present Address: Centre for Research in Agricultural Genomics (CRAG), Consortium CSIC-IRTA-UAB-UB, Edifici CRAG, Campus Universitat Autonoma Barcelona, Bellaterra, 08193 Spain; Genètica i Millora Animal, IRTA, Av. Alcalde Rovira Roure, 191, Lleida, 25198 Spain; Institut Català de Recerca i Estudis Avançats (ICREA), Barcelona, 08010 Spain

**Keywords:** Porcine linkage maps, Recombination, X chromosome, European and Asian X chromosome

## Abstract

**Background:**

Linkage maps are essential tools for the study of several topics in genome biology. High density linkage maps for the porcine autosomes have been constructed exploiting the high density data provided by the PorcineSNP60 BeadChip. However, a high density SSCX linkage map has not been reported up to date. The aim of the current study was to build an accurate linkage map of SSCX to provide precise estimates of recombination rates along this chromosome and creating a new tool for QTL fine mapping.

**Results:**

A female-specific high density linkage map was built for SSCX using *Sscrofa10.2* annotation. The total length of this chromosome was 84.61 cM; although the average recombination rate was 0.60 cM/Mb, both cold and hot recombination regions were identified. A Bayesian probabilistic to genetic groups and revealed that the animals used in the current study for linkage map construction were likely to be carriers of X chromosomes of European origin. Finally, the newly generated linkage map was used to fine-map a QTL at 16 cM for intramuscular fat content (IMF) measured on *longissimus dorsi*. The *sulfatase isozyme S* gene constitutes a functional and positional candidate gene underlying the QTL effect*.*

**Conclusions:**

The current study presents for the first time a high density linkage map for SSCX and supports the presence of cold and hot recombination intervals along this chromosome. The large cold recombination region in the central segment of the chromosome is not likely to be due to structural differences between X chromosomes of European and Asian origin. In addition, the newly generated linkage map has allowed us to fine-map a QTL on SSCX for fat deposition.

**Electronic supplementary material:**

The online version of this article (doi:10.1186/s12863-014-0148-x) contains supplementary material, which is available to authorized users.

## Background

Linkage maps are key tools to genetically map and dissect complex traits, as well as for the study of several topics in genome biology such as the molecular basis of recombination and evolutionary genomics [1]. Interestingly, previous studies have reported larger recombination rate variations across and within chromosomes from swine species than those observed in other mammals [2]. These and other results, such as the construction of the most recent porcine linkage maps, have been enabled by the high density of markers provided by the PorcineSNP60 BeadChip [3,4].

The X chromosome plays an important role in the evolution of human and animals [5], and experiences higher selection pressure than autosomes due to the sex-specific dosage compensation [6]. Moreover, the X chromosome of pigs carries many interesting genes involved in development, fertility, reproduction and diseases such as the *inactive X specific transcripts (XIST)*, *androgen receptor (AR)* and *thyroid-binding globulin (TGB)*, and over 370 QTLs for productive and reproductive related traits have been reported on this chromosome (www.animalgenome.org/cgi-bin/QTLdb). However, the location of these QTL is not precise, due to the low density of the available linkage map. In spite of its relevance, the highest density linkage map for the porcine X chromosome to date includes only 60 markers [7]. None of the above mentioned high density linkage maps include this chromosome.

High density genetic linkage maps are not only essential for QTL fine-mapping, they are also needed to successfully identify functional and positional candidate genes that may carry causal mutations. Therefore, the aim of the current study was to construct a high density linkage map of the SSCX, obtaining precise estimates of the recombination rate along this chromosome. Furthermore, we have employed the new dense marker linkage map to identify possible QTL for several production and meat quality traits in an experimental Iberian x Landrace cross.

## Methods

### Linkage map construction

The animals used in the current study belong to three generations of an experimental Iberian x Landrace cross, the so-called IBMAP pedigree [3]. Briefly, there were a total of 416 pigs of the IBMAP experimental cross, comprising 147 males and 269 females organized in 62 families. There were 86 F3 animals from the cross of three F2 boars with 15 F2 sows, 79 backcrossed animals (BC2) from the cross of four F2 boars with 22 Landrace sows, and 160 backcrossed animals (BC1) from the cross of five F1 boars with 25 Landrace sows. In addition, F1 and F0 sires and dams of the F2 and F1 animals described were also genotyped. A total of 329 meiotic events were available for further analyses. Animal manipulations were performed according to the Spanish Policy for Animal Protection RD1201/05, which meets the European Union Directive 86/609 about the protection of animals used in experimentation. The animals were genotyped with the PorcineSNP60 BeadChip [8] using the Infinium HD Assay Ultra protocol (Illumina, Inc.). Raw individual data had high-genotyping quality (call rate >0.99). The clustering of the genotype data obtained with the Illumina BeadStudio software was checked, and markers with poor clustering performance (GenScore <0.85) were excluded from the analysis. The high-quality SNPs mapped on SSCX following *Sscrofa10.2* genome assembly [9] were retained, giving a total of 426 SNPs that were used for further analyses.

The linkage map was built employing exclusively the female genotypes and using those high-quality SNPs with a minor allele frequency higher than 0.15 (a total of 200 SNPs) (Additional file 1: Table S1). The Fixed option of the updated CRI-MAP v2.503 (provided by JF Maddox, http://www.animalgenome.org/bioinfo/tools/share/crimap/) was used for linkage map construction. The order given to the SNPs followed the physical order of the *Sscrofa10.2* assembly. Note that possible errors in the Xq tail assembly (from 125 Mb in *Sscrofa9* version corresponding to 144 Mb in *Sscrofa10.2* version) have been reported [10]. However, the SNP data used in the current study span from 0 to 143 Mb, therefore the assembly region containing potential errors is not included in the analyses. In an attempt to evaluate remaining genotyping errors and mapping mistakes, Chrompic option of CRI-MAP was employed to reconstruct the chromosomes and carefully check double crossovers. The average recombination rates were calculated as the ratio between genetic and physical lengths (cM/Mb) from the first to the last marker. Genetic vs. physical distances were plotted considering the exact SNP linkage (cM) and physical (Mb) positions.

Correlations between recombination rate and SNP number, GC content (%GC) and gene content were examined. The SNP number, %GC and gene content were calculated along the X chromosome in 1 Mb non-overlapping windows. The gene content was estimated using the porcine genome annotation *Sscrofa10.2* in BioMart tool of Ensembl (ensembl.org/biomart) [11] and the Ensembl Genes 76 database.

### Chromosome X origin

To infer the origins of the chromosome X segregating in the mentioned IBMAP experimental cross, 100 animals with available genotypes from the Porcine60SNP BeadChip [10] and the 79 parental pigs of the experimental cross were included in the analysis. These animals were grouped into five pre-defined populations: 21 Asian wild boars from Korea (5) and Russia (16), 52 Asian domestic pigs from Korea (9) and China (43), 17 European wild boars from Poland (8), Tunisia (8) and Hungary (1), 13 Iberian pigs including the 3 sires and the 76 Landrace dams that were founders of the experimental cross. The genotypes for the 426 high-quality SNPs were extracted from the whole SNP dataset.

The SNPs contained within the pseudoautosomal (PAR) and non-pseudoautosomal (NPAR) regions of X chromosome were differentiated following Burgos-Paz et al. [12] criteria. The first 37 SNPs (from 0 to 6.54 Mb) fall within PAR and the remaining 389 (from 7.18 to 143.48 Mb) within NPAR, and each one of these chromosome regions was independently analyzed. A Bayesian clustering method in STRUCTURE software [13] was used to assign individuals to one of the K clusters representing ancestral populations, or jointly to two or more populations if their genotypes indicated that they were admixed. In this analysis, an admixed ancestry model was assumed, and K values equal to 2 and 3 were considered according to the results reported in the quoted study concerning worldwide genetic relationships between pigs based on SSCX SNPs [12]. For each analysis performed, a burn-in period of 50,000 iterations was followed by 500,000 iterations.

### QTL scan

A QTL scan on SSCX was conducted for growth, fatness, intramuscular fat content (IMF) and body conformation traits recorded on 134 fattened pigs from the F3 (*n* = 55) and BC2 (*n* = 79) generations of the IBMAP experimental cross. The BC1 pigs were not included in the QTL scan as the segregating X chromosomes were exclusively of Landrace origin (F1 boars (♂ Iberian x ♀ Landrace) x Landrace sows). A summary of the phenotypic traits analyzed is shown in Table 1.

**Table 1:** **Phenotypic traits recorded in 134 fattened pigs from the IBMAP intercross used for QTL detection on SSCX**

**Description**	**Trait**	**Mean**	**SD**
Weight at 150 days (kg)	W150d	81.59	12.99
Backfat thickness at slaughter (cm)	BFTS	2.24	0.45
Intramuscular fat content (%)	IMF	1.05	0.54
Mean weight of hams (kg)	HW	11.57	1.68
Mean weight of shoulders (kg)	SW	4.61	0.69

SD: Standard Deviations.

The QTL scan was performed with the following basic model:

$$ {y}_{ijk}={S}_i+{B}_j+{u}_k+b{x}_k+{P}_{ak}a+{e}_{ijk} $$

where *y*_*ijk*_ is the *ijk*^th^ observation for the analyzed trait, *S*_i_ and *B*_j_ are the systematic effects for sex (male or female) and generation/cross (three levels), *u*_k_ is the random polygenic effect of the *k*^th^ individual, *x*_k_ is a covariable (individual age, body or carcass weight in different analyses) and *b* its respective slope, *a* is the QTL additive effect; *P*_*ak*_ is the probability of the *k*^th^ individual having an allele of Iberian and *e*_ijk_ is the random residual. The probabilities *P* were obtained using a modification of the MCMC algorithm described by Pérez-Enciso et al. [14] allowing for the fact that females have two X chromosomes and males have one, and including a weighting factor (Ψ = ½) to account for gene inactivation in females. The infinitesimal genetic effect was treated as random, with covariance *A*σ^2^_u_, *A* being the numerator relationship matrix.

Likelihood ratio tests (LRT) were calculated comparing the full model and a reduced model without the corresponding QTL effect. The nominal *P*-values were calculated assuming a χ^2^ distribution of the LRT with the degrees of freedom given by the difference between the number of estimated parameters in the reduced and full models. Note that permutation techniques cannot be used to calculate chromosome-wide *P* with animal models because the pedigree structure is broken. It is assumed that a nominal 10^−3^*P*-value is equivalent to a 1% chromosome-wide level [15]. All the statistical analyses were performed using the Qxpak v.5.1 software [16]. The confidence intervals (CI) were calculated at 95% following Mangin et al. [17]

## Results and discussion

**Figure 1:**
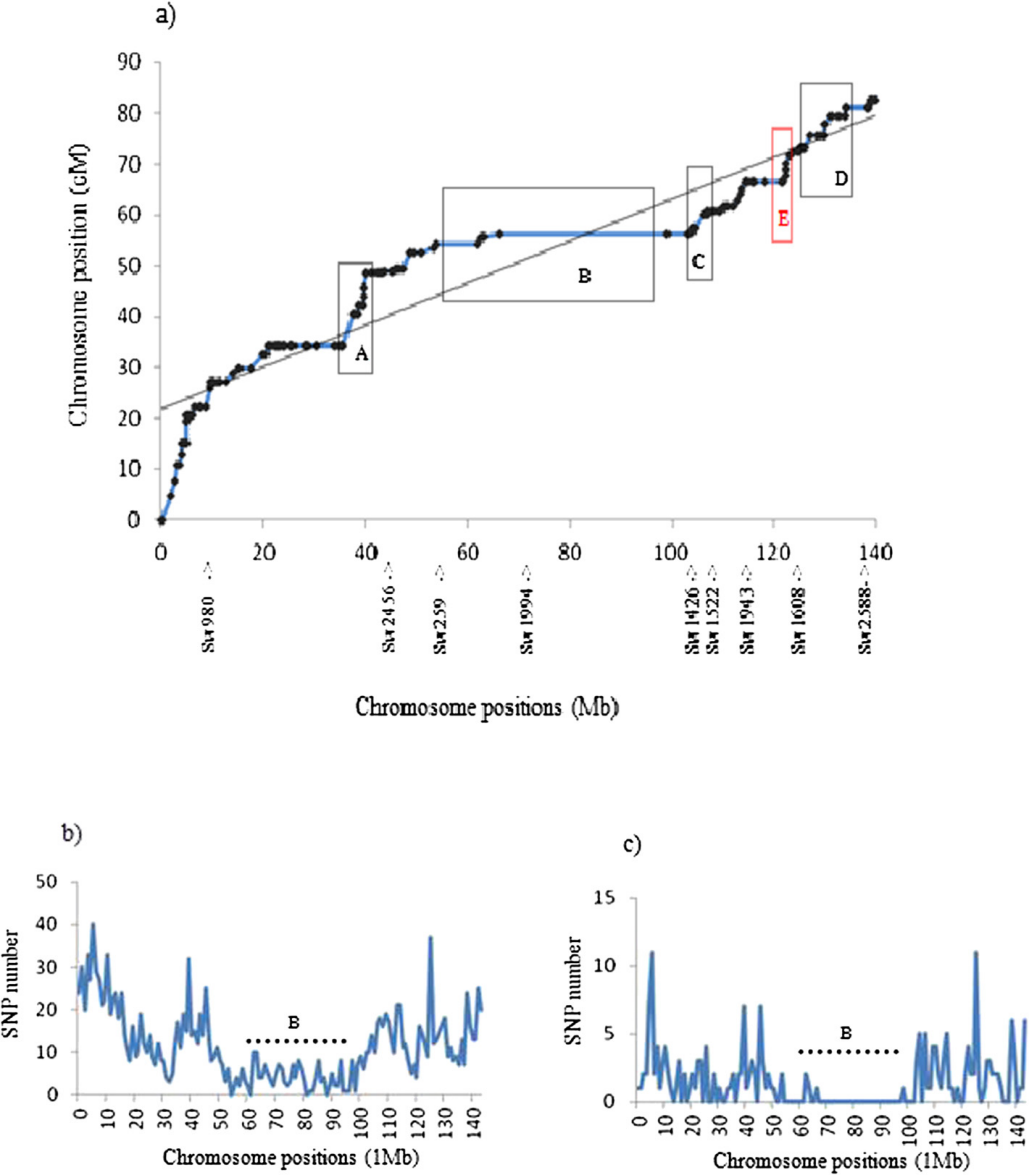
**Physical distance versus linkage distance and SNP number for female-specific SSCX.** **a)** Distribution of genetic (cM) against physical (Mb) distances for female-specific SSCX according to *Sscrofa10.2*. A, B, C, D and E boxes frame cold and hot recombination spots. **b)** Distribution of the SNPs contained in PorcineSNP60 BeadChip and mapped on SSCX according to *Sscrofa10.2*. **c)** Distribution of the SNPs retained in the current dataset and mapped on SSCX.

**Figure 2:**
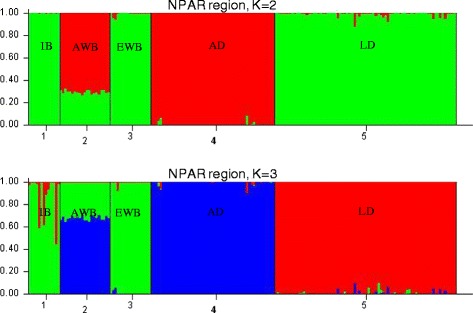
**Average Bayesian probabilistic cluster assignments by population in the non-pseudoautosomal region (K = 2 and K = 3).** IB = Iberian; AWB = Asian wild boar; EWB: European wild boar; AD: Asian domestic pigs; LD: Landrace.

A female-specific high density linkage map was built for chromosome SSCX using *Sscrofa10.2* annotation (Additional file 2: Table S2). The total length of this chromosome was 84.61 cM, shorter than the three SSCX lengths previously reported by Ma et al. [7] (111.4, 159.7 and 128.4 cM), with an average recombination rate of 0.60 cM/Mb. To visualize the results, genetic vs. physical distances were plotted (Figure 1a). Although the total genetic length differs between Ma et al. [7] study and the present one, similar recombination cold and hot regions along SSCX were identified. The markers used in Ma et al. [7] study were annotated here using *Sscrofa10.2* assembly version in order to compare SSCX regions. The central section of the chromosome (B interval from 62 to 103 Mb), comprising the centromere, showed almost no recombination events (average recombination rate = 0.05 cM/Mb). Note that few useful SNPs were available in this interval not only in the selected set for the analyzed population (Figure 1c), but also in the whole PorcineSNP60 BeadChip (Figure 1b) [18]. This large cold recombination section was previously described in Ma et al. [7]. That study was based on two F2 crosses between Western and Chinese pigs; therefore it could be hypothesized that the recombination suppression in such regions might be due to structural differences (insertions, deletions, translocations) between Asian and Western X chromosomes. This hypothesis has been refuted in the present study by determining the X chromosome origin. The results of the analysis conducted to determine whether the NPAR X chromosomes of the animals used in the current linkage mapping were of Asian or European origin are shown in Figure 2. Assuming two ancestral populations (K = 2), the European wild boars, Iberian and Landrace pigs clustered in the same genetic group with a membership proportion higher than 0.99. A similar proportion of Asian domestic pigs clustered in a separate group, and the analyzed Asian wild boars displayed admixture of European and Asian genetic components with averaged individual proportions of 0.30 and 0.70, respectively (Figure 2). Results from the alternative scenario (K = 3), with the assumption of two population clusters of European origin [11], confirm the separate and admixed clustering of Asian pigs and wild boars, respectively. Whereas Iberian pigs and European wild boars presented some admixture of the other two population clusters, Landrace pigs clustered in one of these genetic groups with an average proportion higher than 0.99. The Iberian and Landrace parents of the intercross are likely to be carriers of European X chromosomes rather than Asian X chromosomes, although there were probably two different X chromosome of European origin, according to the probabilistic assignments of individuals obtained when K = 3 (Figure 2). Both NPAR (Figure 2) and PAR (Additional file 3: Figure S1) analyses revealed similar results, with minor differences attributable to difference in the number of SNPs analyzed which was very low in the latter. Therefore structural differences between Asian and European X chromosomes would not provide a plausible explanation for the recombination suppression in the B interval, in agreement with other results where a remarkable homozygosity is observed in this section of the chromosome across European and Asian pigs and wild boars [19,20].

Conversely, four other X chromosome intervals showed recombination rates much higher than the average: the A interval ranging from 35 to 40 Mb, C interval from 103 to 106 Mb, D interval from 130 to 140 Mb and E interval from 121 to 123 Mb, showed recombination rates of 3.17, 1.42, 0.75 and 4.02 cM/Mb, respectively. Apart from the E interval, all of them correspond to the same recombination hotspots identified by Ma et al. [7] with slight differences. The E interval, containing six SNPs, revealed the largest recombination rate along SSCX, and it had not been identified in the previous study likely due to the lack of available markers. It should be noted that SSCX 10.2 assembly has several gaps, and some genome regions are missing. In fact the A region presents 16 gaps, the C region 8 gaps, the D region 19 gaps and the E region 3 gaps. Therefore it is possible that the physical lengths are larger than those used for recombination rate estimates.

In order to analyze the chromosome features influencing recombination rate on the X chromosome, the correlations between recombination rate and SNP number, GC content (%GC) and gene content were calculated. The correlation between recombination rate and SNP number was −0.14 (*P*-value = 0.12), recombination rate and % GC was 0.20 (*P*-value = 0.04) and recombination rate and gene content was 0.01 (*P*-value = 0.42). The only chromosomal feature significantly correlated with the recombination rate was to the GC content. The regions displaying higher recombination rates tended to show enriched GC sequences, in agreement with the tendency observed in autosomes [4].

To show an application of the map, a QTL scan for diverse traits in the experimental Iberian x Landrace cross was conducted using the newly generated linkage map for SSCX containing 200 SNP markers. The results of the analysis are shown in Table 2. The analysis revealed a QTL at 16 cM for the intramuscular fat content of *longissimus dorsi* with an additive effect of 0.33 ± 0.12% and a nominal *P*-value close to the 1% chromosome-wide level. Previous studies on the IBMAP population based on few microsatellite markers revealed a significant QTL in SSCX for live weight before slaughter [15]. However this result could not be validated in the current study, based on animals from other generations of this intercross. A QTL for IMF was previously reported on SSCX by Ma et al. [21] in two large-scale F2 intercosses between breeds with some alleles of Asian ancestry. Nevertheless the QTL (59–84 cM, 42–108 Mb) mapped far from the one identified in the present study.

**Table 2:** **QTL detection for the analyzed traits: Positions, confidence intervals and additive effects**

**Trait**	**Position (CI)**	**LRT**	***a*** **(SE)**	**Nominal** ***P*** **-values**
W150d	47	2.23	4.69 (3.12)	0.14
BFTS	21	3.19	−0.23 (0.13)	0.07
IMF	16 (13–20)	9.74	0.33 (0.12)	0.002
HW	15	2.11	−0.41 (0.27)	0.15
SW	85	2.06	0.45 (0.28)	0.15

W150d: Weight at 150 days (kg); BFTS: Backfat thickness at slaughter (cm); IMF: Intramuscular fat content (%); HW: Mean weight of hams (kg); SW: Mean weight of shoulders (kg). Position in cM, CI: confidence interval; LTR: Likelihood ratio test values; *a* (SE): additive effect (standard error).

The confidence interval of the QTL for IMF was 13–20 cM, which corresponds to 3.5-5 Mb. The QTL falls within the PAR and outside of the cold and hot recombination intervals. This region contains only three protein-coding genes, including an interesting candidate gene for fat deposition, which require further investigation, the *steroid sulfatase isozyme S* (*STS*), located at 4.1 Mb on SSCX and involved in energy metabolism regulation and insulin sensitivity [22].

## Conclusions

The present study presents for the first time a high density linkage map for SSCX and supports the existence of cold and hot recombination regions. The large cold recombination region in the central segment of the chromosome is not likely to be due to structural differences between Asian and European X chromosomes as previously hypothesized. The new generated linkage map has allowed us to identify a QTL for IMF content, an important meat quality parameter.

## Additional files

Additional file 1: Table S1 SNPs used for the X linkage map construction. Additional file 2: Table S2 X chromosome linkage map. Additional file 3: Figure S1 Average Bayesian probabilistic cluster assignments by population in the pseudoautosomal region (K = 2 and K = 3). IB: Iberian; AWB: Asian wild boar; EWB: European wild boar; AD: Asian domestic pigs; LD: Landrace.

## Abbreviations

QTL: Quantitative trait loci
SNP: Single nucleotide polymorphism
SSC: Sus scrofa chromosome
cM: centiMorgan
Mb: Megabase
LRT: Likelihood ratio test

## References

[1] Arnheim N, Calabrese P, Nordborg M (2003). Hot and cold spots of recombination in the human genome: the reason we should find them and how this can be achieved. Am J Hum Genet.

[2] Bosse M, Megens HJ, Madsen O, Paudel Y, Frantz LA, Schook LB, Crooijmans RP, Groenen MA: **Regions of homozygosity in the porcine genome: consequence of demography and the recombination landscape.** *PLoS Genet* 2012, **8:**e1003100. 10.1371/journal.pgen.1003100 PMC3510040 23209444

[3] Muñoz M, Alves E, Ramayo-Caldas Y, Casellas J, Rodríguez C, Folch JM, Silió L, Fernández AI (2012). Recombination rates across porcine autosomes inferred from high-density linkage maps. Anim Genet.

[4] Tortereau F, Servin B, Frantz L, Megens HJ, Milan D, Rohrer G, Wiedmann R, Beever J, Archibald AL, Schook LB, Groenen MA: **A high density recombination map of the pig reveals a correlation between sex-specific recombination and GC content.** *BMC Genomics* 2012, **13:**586. 10.1186/1471-2164-13-586 PMC3499283 23152986

[5] McVicker G, Gordon D, Davis C, Green P: **Widespread genomic signatures of natural selection in hominid evolution.** *PLoS Genet* 2009, **5:**e1000471. 10.1371/journal.pgen.1000471 PMC2669884 19424416

[6] Nguyen DK, Disteche CM (2006). Dosage compensation of the active X chromosome in mammals. Nat Genet.

[7] Ma J, Iannuccelli N, Duan Y, Huang W, Guo B, Riquet J, Huang L, Milan D: **Recombinational landscape of porcine X chromosome and individual variation in female meiotic recombination associated with haplotypes of Chinese pigs.** *BMC Genomics* 2010, **11:**15. 10.1186/1471-2164-11-159 PMC2850356 20211033

[8] Ramos AM, Crooijmans RP, Affara NA, Amaral AJ, Archibald AL, Beever JE, Bendixen C, Churcher C, Clark R, Dehais P, Hansen MS, Hedegaard J, Hu ZL, Kerstens HH, Law AS, Megens HJ, Milan D, Nonneman DJ, Rohrer GA, Rothschild MF, Smith TP, Schnabel RD, Van Tassell CP, Taylor JF, Wiedmann RT, Schook LB, Groenen MA: **Design of a high density SNP genotyping assay in the pig using SNPs identified and characterized by next generation sequencing technology.** *PLoS One* 2009, **4:**e6524. 10.1371/journal.pone.0006524 PMC2716536 19654876

[9] Groenen MA, Archibald AL, Uenishi H, Tuggle CK, Takeuchi Y, Rothschild MF, Rogel-Gaillard C, Park C, Milan D, Megens HJ, Li S, Larkin DM, Kim H, Frantz LA, Caccamo M, Ahn H, Aken BL, Anselmo A, Anthon C, Auvil L, Badaoui B, Beattie CW, Bendixen C, Berman D, Blecha F, Blomberg J, Bolund L, Bosse M, Botti S, Bujie Z (2012). Analyses of pig genomes provide insight into porcine demography and evolution. Nature.

[10] Burgos-Paz W, Souza CA, Megens HJ, Ramayo-Caldas Y, Melo M, Lemús-Flores C, Caal E, Soto HW, Martínez R, Alvarez LA, Aguirre L, Iñiguez V, Revidatti MA, Martínez-López OR, Llambi S, Esteve-Codina A, Rodríguez MC, Crooijmans RP, Paiva SR, Schook LB, Groenen MA, Pérez-Enciso M (2013). Porcine colonization of the Americas: a 60 k SNP story. Heredity.

[11] Haider S, Ballester B, Smedley D, Zhang J, Rice P, Kasprzyk A (2009). BioMart Central Portal—unified access to biological data. Nucleic Acids Res.

[12] Burgos-Paz W, Souza CA, Castelló A, Mercadé A, Okumura N, Sheremet’eva IN, Huang LS, Cho IC, Paiva SR, Ramos-Onsins S, Pérez-Enciso M (2013). Worldwide genetic relationships of pigs as inferred from X chromosome SNPs. Anim Genet.

[13] Pritchard JK, Stephens M, Donnelly P (2000). Inference of population structure using multilocus genotype data. Genetics.

[14] Pérez-Enciso M, Clop A, Noguera JL, Ovilo C, Coll A, Folch JM, Babot D, Estany J, Oliver MA, Diaz I, Sánchez A (2000). A QTL on pig chromosome 4 affects fatty acid metabolism: evidence from an Iberian x Landrace intercross. J Anim Sci.

[15] Pérez-Enciso M, Mercadé A, Bidanel JP, Geldermann H, Cepica S, Bartenschlager H, Varona L, Milan D, Folch JM (2005). Large-scale, multibreed, multitrait analyses of quantitative trait loci experiments: the case of porcine X chromosome. J Anim Sci.

[16] Pérez-Enciso M, Misztal I: **Qxpak.5: old mixed model solutions for new genomics problems.** *BMC Bioinformatics* 2011, **12:**202. 10.1186/1471-2105-12-202 PMC3123239 21612630

[17] Mangin B, Goffinet B, Rebaï A (1994). Constructing confidence intervals for QTL location. Genetics.

[18] Amaral AJ, Megens HJ, Kerstens HH, Heuven HC, Dibbits B, Crooijmans RP, den Dunnen JT, Groenen MA: **Application of massive parallel sequencing to whole genome SNP discovery in the porcine genome.** *BMC Genomics* 2009, **10:**374. 10.1186/1471-2164-10-374 PMC2739861 19674453

[19] Rubin CJ, Megens HJ, Martinez Barrio A, Maqbool K, Sayyab S, Schwochow D, Wang C, Carlborg Ö, Jern P, Jørgensen CB, Archibald AL, Fredholm M, Groenen MA, Andersson L (2012). Strong signatures of selection in the domestic pig genome. Proc Natl Acad Sci U S A.

[20] Esteve-Codina A, Paudel Y, Ferretti L, Raineri E, Megens HJ, Silió L, Rodríguez MC, Groenen MA, Ramos-Onsins SE, Pérez-Enciso M: **Dissecting structural and nucleotide genome-wide variation in inbred Iberian pigs.** *BMC Genomics* 2013, **14:**148. 10.1186/1471-2164-14-148 PMC3601988 23497037

[21] Ma J, Gilbert H, Iannuccelli N, Duan Y, Guo B, Huang W, Ma H, Riquet J, Bidanel JP, Huang L, Milan D: **Fine mapping of fatness QTL on porcine chromosome X and analyses of three positional candidate genes.** *BMC Genet* 2013, **14:**46. 10.1186/1471-2156-14-46 PMC3691627 23725562

[22] Jiang M, He J, Kucera H, Gaikwad NW, Zhang B, Xu M, O’Doherty RM, Selcer KW, Xie W (2014). Hepatic overexpression of steroid sulfatase ameliorates mouse models of obesity and type 2 diabetes through sex-specific mechanisms. J Biol Chem.

