# Supplementary figures and images for: Recombination of the porcine X chromosome: a high density linkage map
Source: BMC Genet. 2014 Dec 20;15:148. doi: 10.1186/s12863-014-0148-x (PMC4293812; doi:10.1186/s12863-014-0148-x)

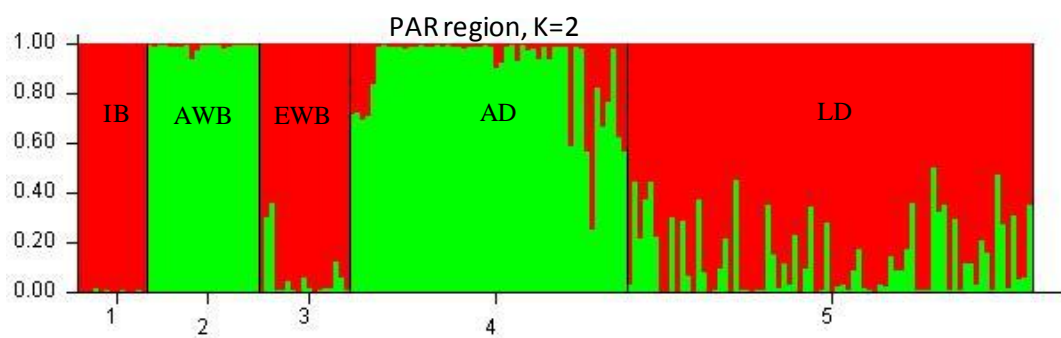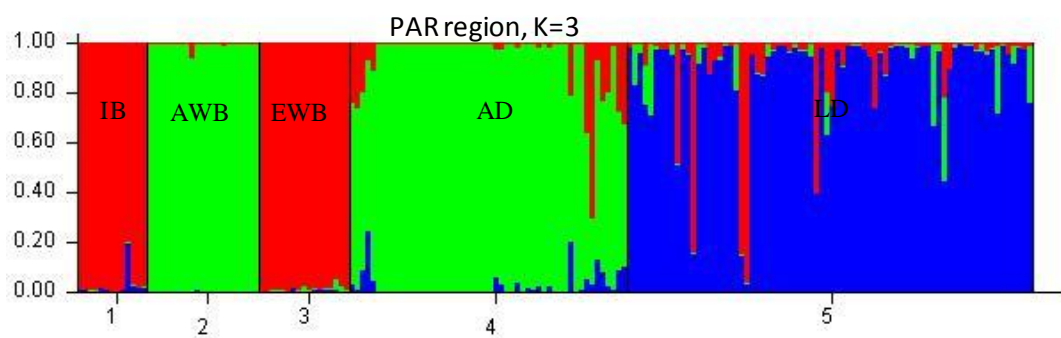

Supplement: Additional file 3: Figure S1 — Average Bayesian probabilistic cluster assignments by population in the pseudoautosomal region (K = 2 and K = 3). IB: Iberian; AWB: Asian wild boar; EWB: European wild boar; AD: Asian domestic pigs; LD: Landrace. [file 12863_2014_148_MOESM3_ESM.pdf]
